# An in-depth exploration of machine learning methods for mental health state detection: a systematic review and analysis

**DOI:** 10.3389/fdgth.2025.1724348

**Published:** 2026-01-02

**Authors:** Md Jawadul Hasan, Shadril Hassan Shifat, Joy Matubber, Rifat Hossain, Md Arifur Rahman, B. M. Taslimul Haque, Md Jakir Hossen

**Affiliations:** 1 ELITE Research Lab, Queens, NY, United States; 2 College of Graduate and Professional Studies, Trine University, Angola, IN, United States; 3 Department of Information Systems, Central Michigan University, Mount Pleasant, MI, United States; 4 Center for Advanced Analytics (CAA), COE for Artificial Intelligence, Faculty of Engineering & Technology (FET) Multimedia University, Melaka, Malaysia

**Keywords:** mental health issues, machine learning, diagnosing, suicidality, social stigma, ethical considerations

## Abstract

**Introduction:**

The global rise in mental health issues has become a significant public health challenge, exacerbated by the reluctance of many individuals to share their mental health concerns due to social stigma. Effective medical interventions and support systems are urgently needed. Researchers are increasingly turning to machine learning as a potential tool for diagnosing and addressing mental health conditions.

**Objective:**

This systematic review identifies and categorizes machine-learning techniques applied to mental health detection, examines studies predicting mental health states, compiles available datasets, and analyzes the most frequently used algorithms for mental health assessment.

**Methods:**

An extensive search was conducted across prominent databases such as Springer, ScienceDirect, IEEE, and PubMed, spanning the period from January 2015 to December 2024, using relevant keywords. Initially, 3,320 articles were selected based on their titles and abstracts. After careful examination, 35 articles met the inclusion criteria. Among the selected 35 studies, 14 leveraged data from online social networks to identify mental health issues, while 21 collected data through various manual means. These studies employed a diverse array of machine learning techniques, encompassing both supervised and unsupervised approaches.

**Results:**

Machine learning exhibits promise in assisting with the diagnosis of mental health conditions and our studies show that machine learning is an effective and efficient way to detect mental health. However, further research is warranted in several key areas. Future studies should explore improved sampling methods, refine prediction algorithms, and address ethical considerations regarding using sensitive mental health data. Furthermore, incorporating image processing techniques could introduce a new dimension to this field. Collaboration with mental health specialists can augment the validity and impact of research outcomes in this critical domain.

**Conclusion:**

The systematic review underscores the potential of machine learning in addressing mental health issues and emphasizes the importance of ongoing research and collaboration to optimize its application in the field. It also shows that, although simpler and more interpretable models such as logistic regression are frequently used as baselines, the highest reported performances are usually achieved by more complex deep learning architectures, underscoring a central trade-off between model interpretability and predictive accuracy in this domain.

## 1 Introduction

Individuals with mental health problems encompass mental illnesses and psychosocial impairments. Mental health patients experience severe distress, difficulty in performing daily activities, or a risk of harming themselves. There is a possibility that people who suffer from psychological disorders have reduced levels of psychological health [1]. Also, the National Institute of Mental Health (NIMH) stated, that roughly 20% of Americans have a mental health issue [2]. In accordance with the WHO, 12.5% of the population is now dealing with a mental health illness, which equates to one out of every eight persons [2]. Even before the COVID-19 epidemic, a tiny fraction of people received proper mental health treatment; for example, only around 12% of people in low-income countries receive treatment for psychosis [3]. According to the World Health Organization, the global population is more susceptible to mental disorders like anxiety, bipolar disorder, depression, eating disorders, schizophrenia, disruptive behavior, post-traumatic stress disorder (PTSD), and dissociative disorders, etc. [4]. In regards to mental health, WHO members signed the “Comprehensive Mental Health Action Plan 2013–2023,” which proposes various measures to prioritize mental health, address related stigma and discrimination, and increase access to treatment. This plan emphasizes the importance of enhancing mental health services, integrating them into primary care, and ensuring human rights for individuals with mental health conditions.

To assess patients’ mental health Traditional methods are time-consuming, costly, and subject to bias. In recent years, researchers have been exploring machine learning (ML) techniques for the detection of mental health and helping healthcare professionals in the treatment process. Text mining and sentiment analysis have become more precise and understandable as a result of the rapid development of machine learning over the past several years [5]. The area of machine learning (ML) focuses on developing and studying algorithms that can learn from and predict future outcomes based on existing data [6]. The ML models are capable of assessing mental health [7]. These ML models need data to be trained, and this data is collected from various sources.

There are currently several review articles on the use of machine learning to predict mental health states. For instance, Liu et al. did a comprehensive review of the literature on depression and social media [8]. There has also been a review that looked at studies that used different methods to check for mental illnesses on social media [9]. Chung et al. conducted a review of various methods for predicting the different states of psychological wellness [10]. Since the widespread adoption of the COVID-19 lockdown, people’s lifestyles have undergone considerable changes, which has had a substantial influence on their mental well-being [7]. Therefore, the key contribution of this paper is mentioned below-

Our study reviewed papers from recent publications that adopted the ML approach to measuring the mental health state. The recent states of mental health and techniques used to predict mental health are discovered in our study.

Our study also reviewed the literature and used information from many different places, such as academic institutions, surveys, and social media, among others.

Our study used 14 articles from online social networks (OSNs) such as Twitter, Facebook, Reddit, and Instagram to find out the large quantities of user-generated data that individuals regularly post, including their thoughts, opinions, and emotions.

Our study found out the machine learning studies of predicting mental health states such as suicide, depression, stress, PTSD, bipolar disorder, anxiety, schizophrenia, ADHD, OCD, eating disorders, Alzheimer’s, Asperger’s, and autism. Our study also helps to find out datasets based on mental health states.

Our study also collects data from other types of online sources, such as pre-made datasets and surveys from people, and our studies also use mental health records and patient data.

Our study described the machine learning and deep learning techniques used for assessing psychological well-being, studied their evolution, assessed their merits and weaknesses, and made recommendations for future studies in this area.

Finally, our study found out the most used machine learning techniques for detecting mental health issues.

However, to fulfill our study’s key contributions followed the proper methodology which is mentioned in Section 2, in Section 3 the result is mentioned and in Section 4 conclusion is written.

## 2 Methodology

**Figure 1:**
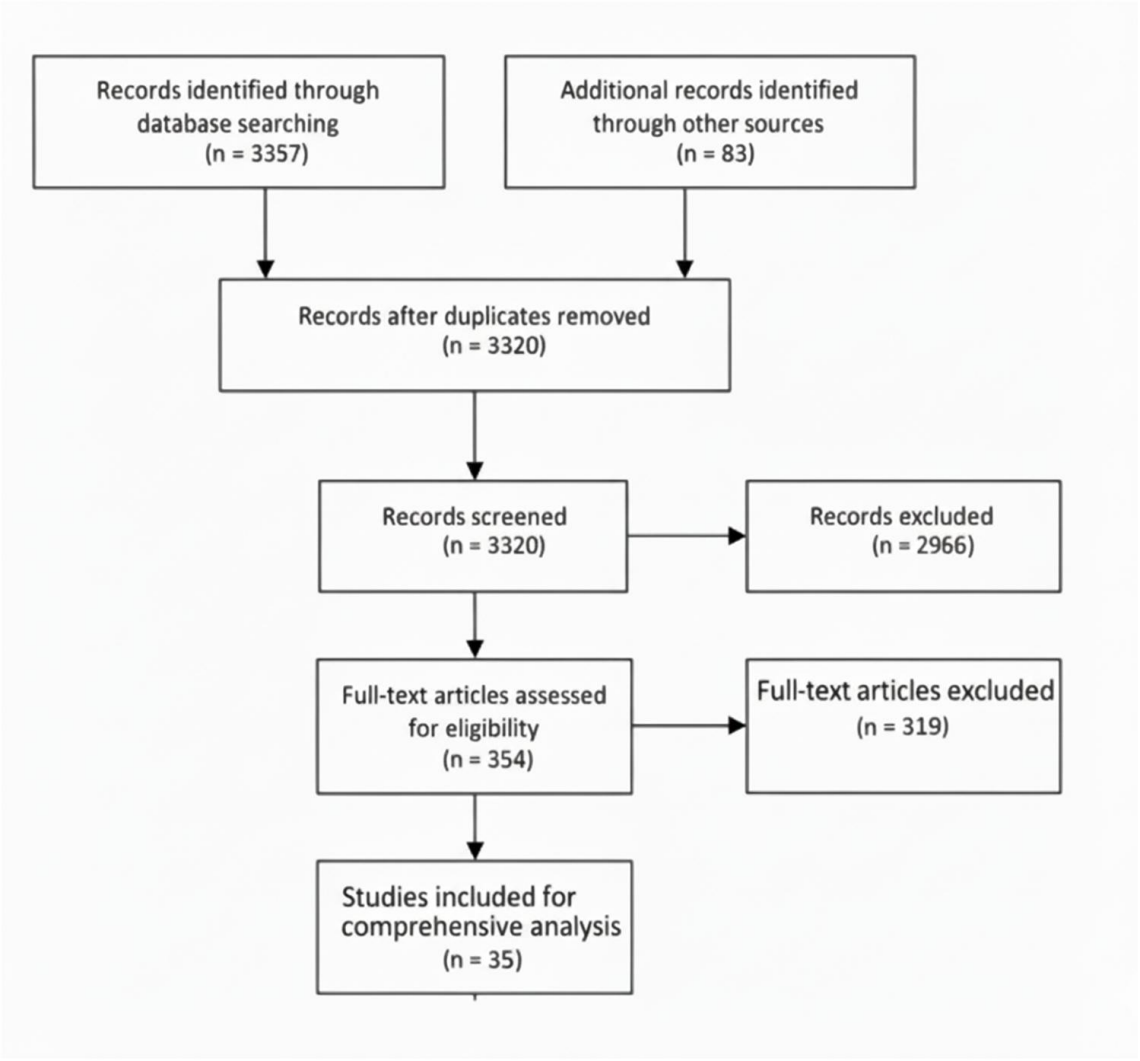
Systematic search flowchart for this review.

This study aims to analyze the efficacy of utilizing machine learning techniques for recognizing mental health, as well as to identify any challenges and limitations that may occur as a result. In accordance with PRISMA (Preferred Reporting Items for Systematic Reviews and Meta-Analyses), this study will follow the framework shown in Figure 1 to systematically document and organize its methodology.

### 2.1 Databases and search keywords

The study included asignificant section of research that employed data from social media, online psychological counseling, and other organizations as a source of data for the identification of mental health concerns. Researchers who used natural language processing techniques to pull out important data from the text and machine learning algorithms to sort and analyze the data were also part of this study. This study comprised research that was obtained from the following libraries, which were used for the research: IEEE Xplore, Scopus, Springer, ACM Digital Library, IGI Global, Science Direct, Aclanthology, Nature, PubMed, and IJECE. In the database libraries, using thesearch keywords “machine learning,” “mental health,” “mental depression,” “social media data,” “anxiety,” “suicide,” “stress” and “big data,” the searches were done in order to find relevant literature. Several libraries needed a few adjustments to the format. Also, the search was conducted in Google Scholar (scholar.google.com) using thesame keywords. Forward search and backward search were also conducted during the search process. A total of 3,440 studies were found using the search keyword after additional filtering based on title and abstract.

The total amount was taken down to 3,320 after we eliminated all of the duplicate articles. After individual evaluation by four reviewers, the total number of articles was dropped to 354. Articles that were released between the years of 2015 and 2024 satisfied the standards for being included. Articles that were not authored in English, were published before 2015, and did not concentrate on the key context in the title are omitted from the overall article count. Articles that do not meet any of the exclusion criteria will be further reviewed based on the availability of their complete article by reviewing the abstract. Additionally, there are certain articles that have been published multiple times in other libraries; such papers are only counted once. Finally, for the comprehensive analyses, exactly 35 articles were selected. Table 1 outlines the criteria for selecting studies, including the inclusion and exclusion standards followed.

**Table 1:** Criteria for inclusion and exclusion.

Inclusion standards	Exclusion standards
Articles published between 2015 and 2024	Articles that don’t address issues with mental health
Articles in English language	Articles that did not meet any of inclusion criteria
Articles that used data from social media, online treatment programs, or various institutions	Articles that are unrelated to data from social media, online treatment programs, or various institutions
Articles employing ML that based on prior research on mental health	Articles with insufficient information
Articles that concentrated the key contexts	Articles that were published before 2015
Journals and relevant conferences emphasize the main theme	Articles that contained duplicated database

## 3 Results

In this systematic literature review, papers are collected from the years 2015–2024. Among the 3,440 primarily collected papers, 35 are selected for this review. The papers are first screened through abstract and title checking, and after meeting the inclusion criteria, these papers were identified as containing the most relevant information about our review topic.

The brief description of the selected articles is given in Table 2, which contains mental health types that are identified using machine learning techniques, dataset information, and the locations and time that the study was conducted. Various deep learning and machine learning algorithms are used in these articles. 14 authors collected their data from different online social media platforms; 12 from different institutions and agencies; and the rest of the papers collected data through conducting surveys. The majority of the authors gathered their data directly from online social media platforms and surveys, while 15 papers re-used data that had been compiled from diverse sources, such as institutions and agencies. The authors [11–25] who used previously existing data showed that deep learning and machine learning can be used to effectively analyze pre-existing datasets.

**Table 2:** Summary of machine learning studies of predicting mental health states.

Authors	Mental health types	Sample	Duration	Geo- locations	Dataset
O’Dea et al. [26]	Suicidality	The authors collected 14,701 suicide-related tweets on Twitter.	Febuary 2014–April 2014	Not-specified	The 1,820 tweets are divided into two groups, each of which is further subdivided into train and test sets.
Lin et al. [27]	Stress	The authors collected 350 million tweets from the Chinese social media platform Sina Weibo.	October 2009–October 2012	China	The data set is separated into two groups: stressed and non-stressed.
Orabi et al. [28]	Depression	The data was discovered through the social media network Twitter.	Not-specified	Not-specified	There were 1,145 Twitter users diagnosed with control, depression, or PTSD. Additionally, each user of the dataset is
Deepali et al. [29]	PTSD, bipolar disorder, anxiety.	The authors collected data of 166 users from twitter.	Not-specified	Not-specified	The 12 lakh tweets are divided into three categories: training, testing, and validation, with 14 features each.
Kim et al. [30]	Depression, anxiety, schizophrenia, bipolar, autism and personality disorder	The authors collected data from reddit based on keyword search.	January 2017–December 2018	Not-specified	After the pre-processing of 633,385 posts, 488,472 posts were used for analysis.
Afzoon et al. [31]	Depression, anxiety, depression+ anxiety	Sentiment140 dataset and Suicide and Depression dataset from reddit is used in this research.	Not-specified	Not-specified	Sentiment140 dataset contains 1,600,000 tweets extracted using the twitter API. Suicide and Depression dataset is collected through Pushshift API
Sekulic et al. [11]	Depression, anxiety, PTSD, autism, OCD schizophrenia, ADHD, bipolar disorder, and eating disorders	The authors collected SMHD dataset which is the collection of Reddit posts from individuals	Not-specified	Not-specified	By creating patterns for recognizing nine distinct self-reported mental illnesses, users were identified.
Raut et al. [12]	Not-specified	The authors used Sentiment140, (MBTI) Myers-Briggs Personality Type Dataset dataset that is collected from kaggle	Not-specified	Not-specified	Along four axes are presented 16 distinct personality types based on intuition, logic, introversion and perceived ability.
Nanath et al. [32]	Not-specified	This research made use of three datasets, the COVID-19 tweets dataset from twitter using twitter API that collected 3 million data, the mobility data from Google, and the lockdown strictness index data from Oxford University	March 2020–April 2020	Not-specified	After exclusion of twitter data Final dataset consist of 150,000 tweets, 2020 google mobility data and the lockdown strictness index data from Oxford University
Hinduja et al. [33]	Depression, schizophrenia, asperger’s, dementia, Alzheimer’s and bipolar disorder	Author collected 2,138 tweets using twitter API that are related to these diseases.	Not-specified	Not-specified	They used binary classification on each of 2,138 tweets and also collected past tweets of 600 users over a month.
Priya et al. [34]	Anxiety, depression and stress	The authors collected data using the DASS-21 questionnaire from 348 participants via google forms	Not-specified	Not-specified	On the Depression, Anxiety, and Stress Scale, 348 observations are labeled as Normal to Extremely Severe.
Tate et al. [35]	Stress, inattention, impulsivity etc	Data collected from 7,638 twins manually	Not-specified	Sweden	85 variables were determined, and the dataset was divided into three parts: the train, tune, and test sets
Karunakaran et al. [36]	Bipolar disorder, anxiety, depression, eating disorder, sleep issues	The authors collected data using two different self-reporting questionnaires.	Not-specified	Not-specified	Out of 1,269 samples, 1,253 valid samples were used for analysis, and 111 people reported having general symptoms of mental issues.
Wang et al. [37]	Not specific	The authors collected data using questionnaire surveys from 5,108 Chinese medical workers.	June 2020	China	The final sample size contains 5,108 data points with 32 variables
Sau et al. [38]	Depression and anxiety	The author collected data set of 470 seafarers.	January 2016–August 2016.	India	The dataset contained 470 observations as well as 14 predictors and ten categorical features.
Ahuja et al. [13]	Stress	The Author collected 206 data from Jaypee Institute of Technology.	Not-specified	India	14 questions were asked of the students and were divided into three categories using weighted average methodology.
Wang et al. [14]	Not specific	They collected data from Nanjing Drum Tower Hospital and Southeast University’s Zhongda Hospital	July 2012–Febuary 2019	China	The dataset contains medical records
Shetty et al. [15]	Depression	They collected Data set from Kaggle dataset on twitter.	Not-specified	India	The suggested LSTM was developed and validated utilizing depression-related tweets from a Kaggle dataset
Perez-Valero et al. [39]	Stress	Offline EEG processing was conducted on the participants’ to collected data.	Not-specified	Spain	Twenty healthy individuals with a mean age of 24.20 ± 4.03 (14 men and 6 women) took part in the study.
Srividya et al. [40]	Not specific	They collected the data through a survey	Not-specified	Not-specified	Among the response from 656, the replies gathered are regarded as data points, and the 20 questions are regarded as features
Sarker et al. [16]	Mental depression	Dataset from Kaggle used in this study.	Not-specified	Not-specified	The dataset contains 2,132 rows and 2,549 columns from the EEG Brainwave Dataset
Dheeraj et al. [17]	Anxiety, insomnia, OCD, stress addiction, depression	Data are collected from Webmd and a online medical healthcare platform Healthtap	Not-specified	Not-specified	The datasets Webmd and Healthtap, including 2,086 and 5,328 questions, respectively, were employed for the deployment of many deep learning models.
Le et al. [41]	Concentrate, distract	The authors selected different representations for facial video data.	Not-specified	Not-specified	Three facial data representations were chosen by the authors: altered videos, landmarks, and embedding vectors
Guo et al. [42]	Not specific	The authors collected data using LMS and questionnaire surveys from 509 Chinese university students	Not-specified	China	Among the 509 university students 485 students are Finally selected after removing the error data.
Liu [18]	Not specific	Microblog data from the COAE2014 conference and IMDB movie reviews data.	Not-specified	Not-specified	12,500 samples from the IMDB dataset, both positive and negative, are used in this study as training sets. The COAE2014 dataset consists of 10,000 manually annotated microblog comment.
Shickel et al. [19]	Positive and negative	The authors collected dataset from TAO Connect1 online therapy program and also used Sentiment1402 public dataset.	Not-specified	Not-specified	3,872 IRB-approved responses from TAO Connect1 online therapy labels were applied to textual replies by three professional psychology graduates under professor supervision
Liu et al. [20]	Bipolar disorder	The author collected 955 samples from mental health center	June 2016–May 2019	Western Canada	Utilizing leave-one-out cross-validation, the final dataset comprised 10 significant features
Madan et al. [21]	Not specific	From 30,000 documents collected from German University Hospital Aachen (UKAachen)	2014–2019	Germany	100 randomly annotated data were chosen for training the model and 50 were chosen for testing
Shickel et al. [22]	Distortions	Author’s collected datasets from Crowdsourced cognitive distortions dataset and TAO connect mental health records.	Not-specified	Not-specified	The crowdsourcing dataset had 7,668 text responses. Also mental health therapy log has 2 subsets, first one was labeled with 15 distortions and second one labeled with binary classification of distortion 0 and 1.
Wang et al. [23]	Pregnant women’s anxiety, depression, hypomania	The Author uses an NHS mental health dataset that includes 223 samples.	Not-specified	Not-specified	From 223 initial samples they got 31 features.
Laijawala et al. [24]	Not specific	OSMI (Open Sourcing Mental Illness) survey dataset was used.	Not-specified	Not-specified	Final dataset consists of 26 attributes and 1 label.
de Lacy et al. [25]	Anxiety, depression, attention deficit, disruptive behaviors, post-traumatic stress (PTS)	All data are collected by the Child Mind Study Team as part of the ongoing Healthy Brain Network (HBN) study.	Not-specified	USA	The dataset comprises 1,120 adolescents (729 male, 391 female). The Edinburgh Handedness Inventory (−100 left to +100 right), FSIQ, autism traits, and cognition were assessed using the WISC-V, ASSQ, card sorting (set shifting), flanker (inhibitory control), and list sorting (working memory).
Oliveira et al. [43]	Anxiety, depression	Portuguese-language Twitter users from the SetembroBR corpus: 18,819 unique users (Diagnosed vs. 7 × larger Control), 46.8M tweets; features include friends, followers, and mentions	Not-specified	Brazil	SetembroBR social media corpus with textual timelines + non-textual connections
Abdurrahim et al. [44]	Borderline personality disorder (BPD), anxiety, depression, bipolar disorder, mental illness, schizophrenia, poison	Reddit/Kaggle text posts; 7-class setup.	Not-specified	Indonesia	Mental Health Corpus (Kaggle) + Reddit Mental Disorders Identification (Kaggle); Training set: 35,000 records (≈5,000 per label); Test set: 6,108 records
Alqazzaz et al. [45]	Depression (primary; Twitter emotional states such as anxiety, sadness, anger also analyzed)	Twitter posts collected via keyword/lexicon scraping plus limited manual collection; total ≈36,587 tweets; standard text cleaning, bigram tokenization; dataset attributes include user, location, URL, hashtags, mentions, symbols	Not-Specified	Saudi Arabia	Public Twitter data obtained via lexicon keywords (e.g., depressed, suicide, anxiety, hopeless); internally compiled corpus described by the authors

### 3.1 Description of selected studies

The major findings and summary of the papers, which include the objectives, machine learning methods, algorithms, and model performance, are presented in Table 3. The authors analyzed different types of mental health. Depression was studied in the 16 studies [11, 15–17, 23, 25, 28, 30, 31, 33, 34, 36, 38, 43–45]. Meanwhile, stress was investigated in 6 studies [13, 17, 27, 34, 35, 39]. Suicide is studied in only one study [26]. The majority of the articles explored several mental health conditions in their single studies. Sekulic et al. Investigated Bipolar disorder, Anxiety, Depression, ADHD, PTSD, Autism, OCD, and eating disorders in his study. He used the SMHD dataset, which is a collection of Reddit posts from individuals. The dataset is labeled with these nine mental health types [11]. In total, 12 studies [11, 17, 23, 29–31, 33–36, 38, 41] investigated various types of mental health. Meanwhile, no specific mental health types were mentioned in the 9 studies [12, 14, 19, 21, 24, 32, 37, 40, 42]. These studies were carried out in countries such as China, Sweden, India, Spain, western Canada, and Germany. The geo-locations of the studies were specified by 15 authors. China was investigated in 4 studies [14, 27, 37, 42]. In contrast, India was the subject of three studies [13, 15, 38]. Only one study per country was conducted in Germany [21], Western Canada [20], Spain [39], Sweden [35], USA [25], Brazil [43], Indonesia [44], and Saudi Arabia [45]. The majority of the studies are on general populations, but some authors conducted their research on specific target populations such as students, children, and patients.

**Table 3:** Summary of machine learning classifier performance.

Authors	Objectives	ML techniques	Performance
O’Dea et al. [26]	This research examined the suicidal tendency based on the analysis of users’ Twitter posts.	LR & SVM	The best-performing algorithm is found to be SVM with TF-IDF and no filtering.
Lin et al. [27]	Based on how people interact on social media, this study came up with a model for figuring out if someone is stressed.	LR, SVM, DNN, RF Hybrid Gradient Boosted DT	The hybrid model with FGM+ on CNN has given the best accuracy.
Orabi et al. [28]	The author of this study compared and contrasted the evaluation of popular deep learning models for identifying user-level depression from tweets.	CNN & RNN	RNN was selected because it achieved highest accuracy of 91.425%
Deepali et al. [29]	This study proposed a model for the detection and recognition of mental health using social media data.	Ensemble, Random forest, Gradient boosting, Ada boosting, Logistic regression, K-neighbors, MLP classifier	The ensemble classifier outperforms the others in terms of accuracy.
Kim et al. [30]	This study came up with a model for figuring out people’s mental health based on what they post on social media and how they act.	XGBoost & convolutional neural network	Accuracy of CNN was found to be the highest.
Afzoon et al. [31]	This study is conducted to identify people’s depression and anxiety using social media content.	Logistic regression, Ridge, BernouliNB, Gradient boosting, XGB, Random forest.	XGB has performed better and has a high F-score.
Sekulic et al. [11]	For large-scale document categorization on social media, this study used a deep neural network.	LR, Linear SVM, Supervised FastText, HAN	For disorders that affect a fair number of people, a HAN is better than the other models.
Raut et al. [12]	Predicting personality and mental health by comments from web based systems.	Logistic regression, Naïve Bayes, SVM, Random forest, K Neighbors and XGBoost	XGBoost’s classifier predicts 16 distinct personalities with the highest accuracy of 67.68 percent, Mental health can be predicted with 96.05% accuracy using logistic regression.
Nanath et al. [32]	The research examined mental health state due to lockdown and created a mental health index.	Fixed-effects ordinary least squares -(OLS)	The study concludes that decreasing mobility in the workplace, retail, and recreational settings and increasing residential mobility have negatively impacted mental health states.
Hinduja et al. [33]	A framework is made for social sensors like twitter to early detection of mental hearth.	LSTM, SVM	The accuracy of the single dense layer got the highest accuracy of 84.31.
Priya et al. [34]	This research examined the anxiety, stress and depression scale.	DT, Random Forest Naive Bayes, SVM, K-NN	The random forest classifier with the f1 measure has given the best accuracy.
Tate et al. [35]	This research suggests a methodology for early identification of adolescent psychological distress.	Logistic regression XGBoost, Random forest SVM, Neural network	Among others, the random forest model (RF) performed well, with an AUC of 0.739.
Karunakaran et al. [36]	The research outlined a machine learning-based approach for evaluating one’s own mental health.	Logistic regression DT, Naïve Bayes, SVM	Logistic regression is the best option for mental health screening and SVM linear is found to best fo multiple mental disorder screening
Wang et al. [37]	The authors of this study created a novel predictive model to identify the elements that have the most impact on the mental health of healthcare workers.	Stepwise logistic regression, Hybrid improved dragonfly algorithm, Binary bat algorithm, Hyper learning binary dragonfly algorithm etc.	Hyper learning binary dragonfly algorithm has given the best result.
Sau et al. [38]	This research compared the efficacy of several machine learning algorithms for screening seafarers for anxiety and depression.	Naive Bayes, CatBoost, SVM, Logistic regression Random forest,	CatBoost achieves 89.3% accuracy and 89.0% precision.
Ahuja et al. [13]	Determine how stressed-out students are when to use the internet one week before examinations. Also examine these things’ effects on them as well.	Linear regression, Random forest, Naïve Bayes, SVM	SVM has a higher accuracy of 85.71% and with 75% sensitivity and specificity of 100%.
Wang et al. [14]	Deep learning neural networks (DLNNs) and Shapley Additive explanation (SHAP) were used in this study to predict the population-level sickness risk of mental illnesses (MDs) and subsequently to explain the risk factors.	DLNNs, LSTM	NSE has the higher accuracy of 88%,
Shetty et al. [15]	The proposed research aims to predict depression in people by looking at their internet activities.	LSTM & CNN	CNN outperforms the others, with a 95% accuracy rate.
Perez-Valero et al. [39]	The study is conducted to analyze the impact of EEG PSD smoothing in three-level stress categorization and to determine if a two-level stress detector can be used in practice without smoothing.	LR, SVM RF, KNN MLP	In non-smoothing: SVM, RF, MLP are selected because they achieved highest accuracy 0.84 ± 0.02, 0.84 ± 0.03, 0.84 ± 0.02)% In smoothing: SVM are selected because it achieved highest accuracy 0.94%
Srividya et al. [40]	This research aims to identify the mental health status of a specific population using multiple machine-learning algorithms.	DT, RF, Naïve Bayes KNN, LR	RF was selected because it achieved highest accuracy of 90%
Sarker et al. [16]	The authors examined and evaluated the applicability of machine learning and deep learning algorithms for detecting mental Depression using EEG data.	MLP, LR, CNN, LSTM, RNN, SVM	SVM and LR have outperformed when applied to EEG brain wave data to detect mental depression.
Dheeraj et al. [17]	This work proposed the MHA-BCNN model, which outperforms previous attempts at collecting negative text-based emotions. MHA-BCNN utilizes convolutional neural network and bidirectional long-short-term memory.	BiLSTM, LSTM-CNN, GRU-BiLSTM-CNN, BiLSTM-CNN, MHA-BCNN	Accuracy of MHA-BCNN was found to be highest.
Le et al. [41]	This research looked at how well different face data formats and deep architectures worked together. The goal was to find a solution that strikes a fine balance in both prediction accuracy and time efficiency and that can be used in real time.	VGG+1DCNN, LM+1DCNN	VGG descriptors with 1DCNN input blocks achieved the best-performed models.
Guo et al. [42]	This research introduced the CASTLE framework for assessing the mental health of students.	CSATLE, Deepwalk+DNN, MANE+DNN, MOON+SVM, MOON+RF, MOON+GBDT	Accuracy of CASTLE was found to be the highest.
Liu [18]	This study proposes a text sentiment analysis method that combines deep learning with the Bag of Words (CBOW) language model.	NB, SVM, CNN, Proposed D-CNN	The accuracy and F1-score of proposed D-CNN was found to be highest.
Shickel et al. [19]	In this paper, the authors proposed a unique sentiment analysis job based on a two-dimensional valence scheme with four emotion classes.	Logistic regression, RNN, BERT pre-trained on BookCorpus dataset.	Both label-specific measures and overall dataset-wide metrics showed that BERT was the model that performed the best.
Liu et al. [20]	Researchers used Mood disorder and clinical questionnaires to develop a machine learning model that identifies bipolar disorder at an early stage.	ElasticNet	The LOOCV performance of Elastic net had 80.6% balanced accuracy for classifying Bipolar Disorder.
Madan et al. [21]	This research aims to identify psychiatric characteristics in mental health records from Germany.	Pre-trained deep neural network (GermanBERT)	German BERT performed well, getting F1 scores of 86% and 91% respectively.
Shickel et al. [22]	The author created a system for the identification and categorization of cognitive distortions within the Mental Health Text.	Logistic regression-only result shown here, SVM, RF, XGBoost, RNN, CNN, BERT	Logistic regression performed well with a weighted F1 score of 0.88 for detecting cognitive distortion.
Wang et al. [23]	The research aims to analyze pregnant women’s anxiety and depression. Also to support them with Chabot.	SVM	Using SVM, the anxiety and depression accuracy was 93%, 91% respectively.
Laijawala et al. [24]	The study identifies the best-performing algorithm and employs that model in a web application that provides users with the likelihood of having a mental health state depending on their input.	Decision tree, Random forest, Naïve Bayes	For an accuracy of 82.2%, decision tree was the top-performing algorithm.
de Lacy et al. [25]	Predict individual cases across multiple adolescent psychiatric conditions and compare algorithm classes (deep neural networks, tree-based, elastic-net logistic); assess relative value of psychosocial vs. neural features	Deep neural networks, XGBoost (tree-based), Logistic regression with ElasticNet regularization	Held-out test performance reported as high for deep learning across targets (e.g., anxiety AUC ≈ 0.94 with accuracy ≈ 95%, precision ≈ 94%, recall ≈ 95%), outperforming XGBoost (e.g., AUC ≈ 0.61)
Oliveira et al. [43]	Compare text-only vs. non-text (network) features for Portuguese mental-health prediction (depression, anxiety) on Twitter using the SetembroBR corpus.	BERT	BERT outperformed Bag-of-Users. Positive-class F1–Depression: BERT 0.40 vs. Friends 0.32, Followers 0.32, Mentions 0.34; Anxiety: BERT 0.36 vs. Friends 0.30, Followers 0.29, Mentions 0.30.
Abdurrahim et al. [44]	Build a CNN–BiLSTM model (with FastText embeddings) to classify seven mental-health categories from social-media text; compare against a BiLSTM baseline	CNN–BiLSTM classifier, baseline BiLSTM	CNN–BiLSTM + FastText: Accuracy = 85%, F1 = 85%; BiLSTM baseline: Accuracy = 83%, F1 = 83%
Alqazzaz et al. [45]	Build a deep-learning model to detect depression (and related social-cyber psychological signals) from Twitter text; design a data aggregation + preprocessing pipeline and compare against classic ML baselines	CNN–LSTM (with attention), Logistic Regression, SVM, KNN, Decision Tree	CNN–LSTM achieved Accuracy = 86.23%, Precision = 87%, Recall = 83%, F1 = 85%; baselines–LR: Acc 59.8%, F1 59.1%; SVM: Acc 60.6%, F1 75%; KNN: Acc 65.2%, F1 52%; DT: Acc 73%, F1 74%; Lexical: Acc 85%, F1 50%

### 3.2 Dataset descriptions

According to a statistic compiled by Smart Insights [1], in 2022, there will be approximately 4.7 billion social media users and this is growing substantially. 2.29 hours per day are spent using social media on average. People express their feelings and emotions on social media, and it has become their daily routine. It is possible to understand any user’s mental health condition by observing his social media accounts. We can obtain the complete image of a person by mining their social media posts. It provides all the information related to his moods, activities, sleep hours, communications, food habits, thinking styles, loneliness, etc. From our selected studies, 14 authors used social media data to predict different mental health conditions. Twitter is used in the studies [12, 15, 26–29, 32, 33, 43, 45] and the rest of the 3 studies [11, 30, 31, 44] used Reddit data. O’Dea et al. [26] collected data from Twitter and had three mental health researchers examine the tweet data individually and label the data [26]. Nanath et al. [32] used Twitter data along with Google Mobility Data and Lockdown Strictness Data from Oxford University during the COVID period of 2020 [32]. The majority of the studies [26–30, 32, 33] collected social media data using keyword matching. But most of them did not specify the keywords that they used for prepping the dataset. These two studies mention keywords like “I’m feeling down,” “depression,” “die alone,” “sleep forever,” and so on. [26, 31]. Hinduja et al. [33] determined from the collected tweets whether the tweets were sarcastic or not. If the tweets are selected as real, then it proceeds further with the data processing methods [33].

Articles that performed mental health prediction on a targeted population collected data from either surveys or different agencies. Tate et al. [35] collected data from 7,638 twins in Sweden through a telephone interview [35]. 7 articles [13, 15, 24, 34, 36, 37, 42] used questionnaires to collect data from their targeted people in surveys. Two researchers [16, 39] extracted data from EEG data. Sarkar et al. [16] collected data directly from Kaggle; Perez-Valero et al. [39] conducted offline EEG processing on 20 people to collect the data. Sau et al. [38] conducted an interview on 470 seafarers between January to August on 2016 in India [38]. the remaining researchers [14, 17–23, 25] gathered data from various medical centers, which contained patient records.

### 3.3 Machine learning techniques

In today’s prediction-based research, machine learning algorithms play an important role. Researchers are using different classification-based ML algorithms to predict different types of mental health conditions based on the users’ data. The majority of research studies employed different machine learning algorithms, including logistic regression (LR), SVM, random forest (RF), K-Nearest Neighbor,Decision tree (DT), Naïve Bayes (NB), etc. These algorithms are used to classify different mental health states that include depression, bipolar disorder, suicidal tendencies, PTSD, etc. Algorithms performance varies based on datasets. Classical models like SVM or logistic regression do well on small, tabular surveys and provide simple, stable baselines. Whereas, CNN-LSTM fits sequences like EEG or time-ordered posts by learning patterns over time. BERT is strong for text since it understands context and long-range words. Deep learning algorithms such as CNN, RNN, LSTM, and DNN were used in 15 studies [14–19, 21, 22, 25, 28, 30, 33, 41, 42, 44, 45]. Three researchers [19, 21, 43] classified text-based data using a pre-trained BERT model. 7 researchers [12, 22, 27, 29–31, 35] used Boost (extreme Gradient Boosting), which is a free software library that implements the gradient boosting algorithm in a way that works well and quickly. It is a tree-based ensemble method. Table 4 shows the top 5 most used algorithms in our studied articles.

**Table 4:** Most used algorithms.

Algorithm name	Number of papers used	Paper count
LR	[11–13, 16, 19, 22, 25–27, 29, 31, 35–40, 45]	18
SVM	[12, 13, 16, 18, 22, 23, 26, 27, 33–36, 38, 39, 45]	15
RF	[12, 13, 22, 24, 27, 29, 31, 34, 35, 38–40]	12
Naïve Bayes	[12, 13, 24, 34, 36, 38, 40]	7
Gradient boosting DT	[24, 27, 29, 31, 34, 36, 40]	7

## 4 Discussion

The goal of this literature review is to summarize studies that have detected mental health states using data collected from social media and other sources such as agencies or surveys. Except for one [41], all of the articles used text-based data. First, the researchers took the features from the data they had collected and fed them into various machine learning and deep learning algorithms to make predictions. The data that is collected online produces features based on their posts, which contain their emotional feelings. The survey data is extracted based on the score that the surveys have predetermined.

### 4.1 Model interpretability—performance trade-off

Across the included studies, we also observe a consistent gap between the most commonly used algorithms and those that achieve the highest reported performance. Simpler and more interpretable models such as logistic regression, support vector machines, Naïve Bayes, and random forests are frequently adopted as baseline approaches, particularly when working with survey scores or hand-crafted features [12, 13, 32, 36, 37, 39]. In contrast, the best accuracies and F1-scores are usually obtained with deep learning architectures such as convolutional and recurrent neural networks or transformer models like BERT, especially on large text corpora and sequential data. This pattern reflects a fundamental trade-off in mental health informatics: classical models provide transparency that supports clinical interpretation and hypothesis generation, whereas deep models can exploit complex non-linear patterns in high dimensional data at the cost of being closer to a black box. Future work should therefore benchmark deep architectures against simple baselines and incorporate model explanation techniques so that gains in predictive performance do not come at the expense of interpretability and clinical trust.

### 4.2 Methodological limitations and common risk-of-bias patterns

A cross-study assessment of the included articles reveals several recurring methodological limitations and risk-of-bias patterns. Many social media—based studies rely on self-reported diagnoses, keyword heuristics, or other weak labeling strategies, which can introduce label noise and misclassification into the ground truth. Sample sizes are often modest and class distributions highly imbalanced, yet performance is frequently reported only in terms of overall accuracy or AUC, which may overestimate performance on the minority class [20, 29, 33, 34, 40]. In addition, very few studies conduct external or temporal validation on independent datasets or platforms, relying instead on internal cross-validation, so the generalizability of the reported models remains uncertain [16, 25, 36, 39]. Finally, many datasets are drawn from narrow or incomplete populations (for example, a single institution or student group), which limits representativeness and may hide performance disparities across demographic subgroups. These patterns indicate that reported results should be interpreted cautiously and that future work should prioritize stronger labeling procedures, class-sensitive metrics such as F1-score and recall for the positive class, and external validation across diverse populations and data sources.

Several limitations and challenges in the prediction of mental health states reviewed here should be acknowledged. Researchers used online data to predict his suicidal tendency, but they did not measure any offline activities that might improve the prediction model and would have been more effective in achieving their objectives [26]. Using a huge quantity of data from social media can improve the prediction model’s accuracy. And the brief dataset that contains personal information like age and gender will help extract more features [12, 26, 31]. Furthermore, ensuring user authenticity is important because there are many fake accounts on Reddit, Twitter, and Facebook, which may lead to biased results [33]. It can be challenging to achieve a fruitful output with social media data because of the constraints of face-to-face interactive contact and the human-computer connection. It’s important to note that all of the studies presented here used only text data from social media for diagnosing depression. This may have limited their ability to predict accurately. But social media data also contains images, videos, and audio as well. Using this data may increase the performance of machine learning or deep learning models.

When conducting surveys, it is helpful to inquire about personal details, such as family ties, student-teacher relationships, etc., in order to obtain more characteristics and boost the model’s accuracy [42]. Also improving the model is possible through research into additional transformer-based methodologies and the extraction of text-based emotions with temporal variations [11, 17]. Again, collecting data from any specific institution may lead to biased results. Using different institutional data will make the model more reliable [19]. Researchers noted that the study’s examination of individuals’ personal information and health records could raise major privacy and ethical concerns regarding data security. Furthermore, privacy and ethical concerns merited careful consideration during the research process.

## 5 Conclusion

Recent years have witnessed significant advancements in the application of machine learning to address mental health issues, particularly in the past several years. These developments hold great promise for significantly improving the detection and diagnosis of mental health conditions. The primary objective of this systematic review was to conduct a thorough assessment of the identification of mental health issues through machine learning. This study delved into the adequacy of the methods used for diagnosing mental health disorders, scrutinizing both the data evaluation techniques and the associated challenges and limitations. Within this study, this study examined the performance of both deep learning and machine learning algorithms, recognizing their pivotal role in enhancing mental health diagnostics. This study also explored various strategies related to data collection and feature extraction, as these factors are integral to the effectiveness of machine learning techniques in this context. It is essential to acknowledge that this research area is still evolving and faces certain limitations. Therefore, further research is imperative, with a particular focus on refining sample collection methods, enhancing predictive accuracy, evaluating the generalizability of research findings, addressing privacy concerns, and upholding robust research ethics standards. Through continued exploration in these areas, the potential benefits of machine learning in mental health can be further realized and harnessed to improve the well-being of individuals worldwide.

## Data Availability

The original contributions presented in the study are included in the article/Supplementary Material, further inquiries can be directed to the corresponding author.
